# Trends on Microalgae-Fungi Consortia Research: An Alternative for Biofuel Production?

**DOI:** 10.3389/fmicb.2022.903737

**Published:** 2022-05-26

**Authors:** Ana Beatriz Lobo-Moreira, Solange Xavier-Santos, Luciana Damacena-Silva, Samantha Salomão Caramori

**Affiliations:** ^1^Post Graduate Program in Natural Resources of Cerrado, State University of Goiás, Anápolis, Brazil; ^2^Laboratory of Basic, Applied and Mycology and Scientific Dissemination (FungiLab), State University of Goiás, Anápolis, Brazil; ^3^Laboratory of Host-Parasite Interactions, State University of Goiás, Anápolis, Brazil; ^4^Laboratory of Biotechnology, State University of Goiás, Anápolis, Brazil

**Keywords:** phytoplankton, fungus, scientometric, microbial co-cultivation, biotechnology

## Abstract

The utilization of microalgae and fungi on an industrial scale is a challenge for researchers. Based on the question “how fungi have contributed to microalgae research?,” we verified the scientific trends on microalgae-fungi consortia focused on biofuels production by searching for articles on the Web of Science and Scopus databases through the terms “microalgae^*^” or phytoplankton and “fung^*^.” We found 1,452 articles published between 1950 and 2020; since 2006, the publication numbers have increased rapidly. The articles were published in 12 languages, but most were written in English (96.3%). Among 72 countries, China (360 articles), USA (344), and Germany (155) led the publication rank. Among the 10 most-prolific authors, 8 were Chinese, like 5 of the most-productive institutions, whereas the National Cheng Kung University was on the top of the list. The sources that published the most on the subject were: Bioresource Technology (96), PLoS ONE (28), and Science of the Total Environment (26). The keyword analysis emphasized the magnitude of applications in microalgae-fungi consortia research. Confirming this research question, biofuels appeared as a research trend, especially biodiesel, biogas, and related terms like lipid, lipid accumulation, anaerobic digestion, and biogas upgrading. For 70 years, articles have been published, where China and the United States seem to dominate the research scenario, and biodiesel is the main biofuel derived from this consortium. However, microalgae-based biofuel biorefinery is still a bottleneck on an industrial scale. Recent environmental challenges, such as greenhouse gas mitigation, can be a promising field for that microalgae-fungi application.

## Introduction

Microalgae (also referred to as phytoplankton) is a generic term used to determine a group of microscopic photosynthetic unicellular or multicellular algae and cyanobacteria that can convert sunlight, water, and carbon dioxide (CO_2_) into biomass and double their body weight in 24 h (Zabed et al., 2020). They can grow in freshwater, seawater, and under harsh conditions, such as brackish water and wastewater (Serejo et al., 2019). The diversity of microalgae ranges from 50,000 (Alam et al., 2015) to 1 million species (Rumin et al., 2020), and they have been studied for more than 100 years (Spier et al., 2020). Among various human applications, microalgae are used in the medicine and pharmaceutical industry, food and nutrition, wastewater treatment, and as a renewable source of energy (Baicha et al., 2016).

Likewise, fungi comprise the most diversified living organisms Kingdom; being estimated that the funga (Kuhar et al., 2018) is composed of 2.2–3.8 million species (Hawksworth and Lücking, 2017). They can be micro or macroscopic organisms, they are all eukaryotic and heterotrophic, and most of them form structures called hypha, except for yeasts (Alexopoulos et al., 1996). Fungi can form mutualistic and symbiotic consortia with other living organisms (Richards et al., 2017). Humans also exploit fungus, especially in biological systems for food and drugs, and to reduce the environmental impacts of chemical industrial processes (Rai et al., 2021).

Microalgae-fungi co-cultivation has gained emphasis in research and industry once it improves the main bottlenecks and barriers to using microalgae biomass, e.g., upgrading organic compounds concentration and assisting in the harvesting process, considered the most energetic and financially costly steps of microalgae culture (Li et al., 2020). Despite this, co-cultivation appears as an alternative to increasing the manufacturing ecological footprint (Brasil et al., 2017), which is a low-cost and low-energy input and chemical-free harvesting method for biofuel production (Chen et al., 2018; Mathimani and Mallick, 2020). Many researchers have quantified the scientific production of microalgae in the biofuel scenario (Coelho et al., 2014; Azadi et al., 2017; Ma et al., 2018; Konur, 2020a) in science, technology, and medicine (Konur, 2020b) worldwide (Garrido-Cardenas et al., 2018), particularly in Europe (Rumin et al., 2020) and Brazil (Nabout et al., 2015; Moreira, 2021). Still, none of them comprised the consortia with fungi.

Based on these findings, a question was left: How fungi have contributed to microalgae research? The answers to this question can provide an overview of the present published bibliography and the gaps in the research as a tool to spread the current technologies and guide future research. That being said, in this research, we aimed to map the microalgae-fungi consortia research focused on the following: (a) the amount of literature produced and when did the interest start in this consortium culture, (b) the countries, institutions, and authors who have published about co-cultivation and how was the collaboration dynamic, (c) the most-relevant articles published about this consortium, and lastly (d) the enclosed topics by these studies and the trend for biofuel production research.

## Methods

Microalgae-fungi consortia data was obtained in the Web of Science (Thomson Reuters) and Scopus databases. We searched for “microalga^*^” or phytoplankton and “fung^*^” on titles, keywords, and abstracts. The search attempts were limited to *articles* comprising the first publication in each database until 2020. All data were downloaded in June of 2021. Several publications, journals, institutions, countries, and idioms were analyzed using Microsoft Office Excel 2016. Authors, countries, institutions collaboration, and keywords associations were made using Bibliometrix (Aria and Cuccurullo, 2017) for RStudio version 2021.09.0 Build 351 (R Core Team, 2022) and SigmaPlot 12.0 (Systat Software and Inc, 2011).

Amongst the limitations of scientometric research (Andreo-Martínez et al., 2020), bias in results can be due to the database chosen and the option to only analyze articles. For example, microalgae is a generic term commonly used in biotechnology research (Rumin et al., 2020), while phytoplankton is mostly used by studies in ecology (Nabout et al., 2015). In this way, the searching terms also contributed to bias in the result analyses, delimitating the comprehension of the courses that the research is taking in each specific study field (Cheng et al., 2020).

## Results

### Publication Outputs

**Figure 1:**
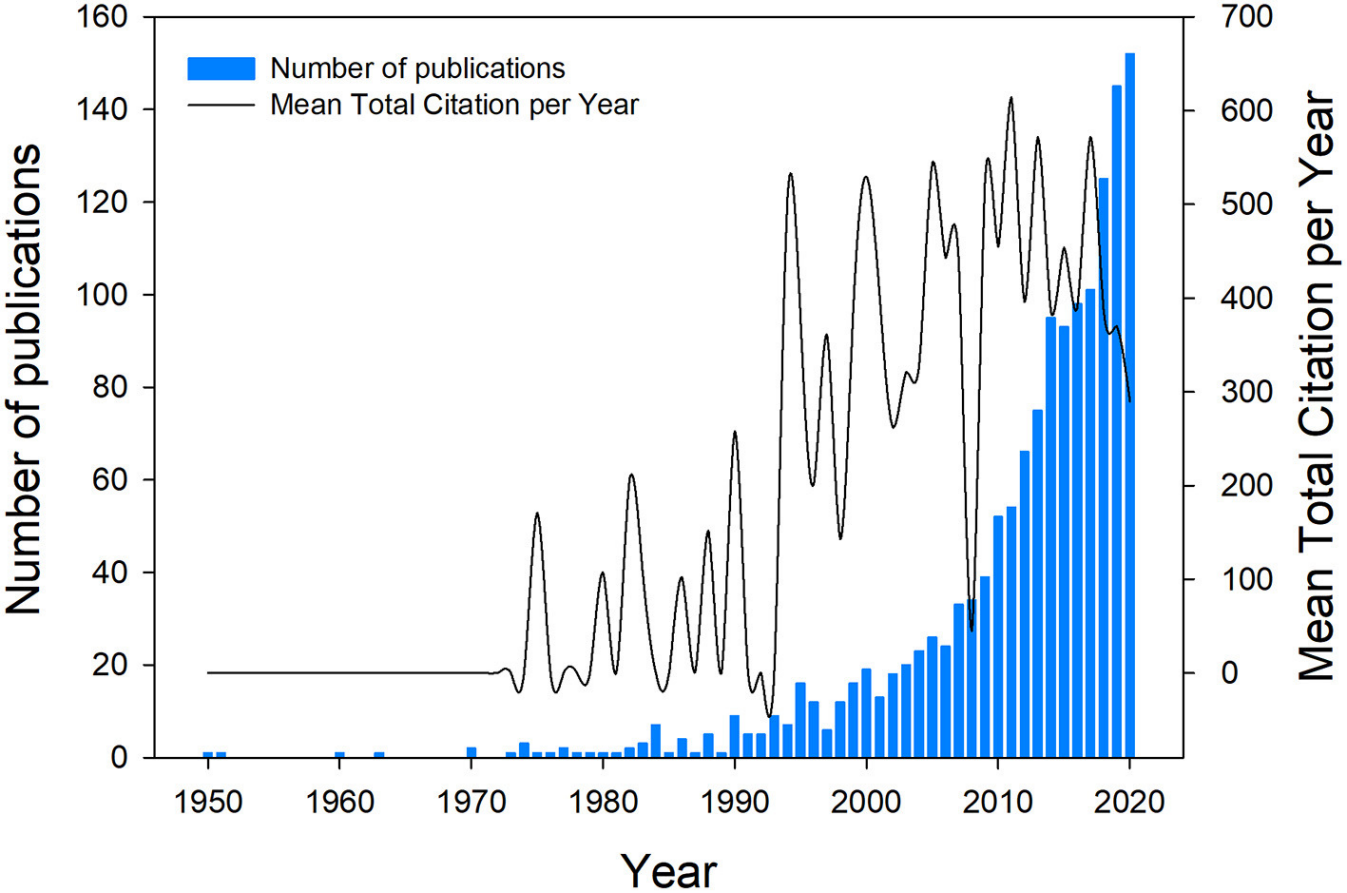
Microalgae-fungi consortia publication and citation outputs among 70 years (1950–2020).

A total of 1,995 articles were downloaded from the two databases, including 902 from the Web of Science and 1,093 in the Scopus database. After removing 543 duplicities, we analyzed about 1,452 articles. The temporal distribution of publication on microalgae-fungi consortia can be observed in Figure 1. The publication increase presented an exponential growth, whereby the average number of publications per year was 20.7 articles.

In reinforcing English as an official scientific publication idiom (Parkinson, 2013), 96.3% of articles were published in English. The remaining 3.7% (53 articles) were published in 11 languages, including German, Chinese, Korean, Spanish, French, Japanese, Persian, Polish, Portuguese, Russian, and Turkish. Citations are also a good measure to quantify the publications. In the case of microalgae-fungi consortia articles, there was a rising tendency in the citation numbers that reached the citation peak in 2010 (Figure 1). Only 6% of the articles did not show citations, while 34.2% showed <10 citations during the time-lapse analyzed. Considering the language, the articles published in English were also the most cited, followed by articles in French (74), Spanish (57), Chinese (52), and Portuguese (32).

All the 1,452 articles summed 49,052 citations, corresponding to an average of 33.7 citations per article. The 10 most-cited articles are described in Table 1, counting 6,021 citations. Confirming the trend for biofuel research using microalgae-fungi consortia, the 3rd and the 6th most-cited articles stated Biodiesel in the title. In the same way, the 2nd and 5th most-cited articles mentioned lipid accumulation, which is one important step in biofuel manufacture.

**Table 1:** The 10 most-cited articles about microalga-fungi consortia.

	**Title**	**Source (Country)**	**Year**	**Citations**
1	Microbial carbonates: the geological record of calcified bacterial algal mats and biofilms	Sedimentology (United Kingdom)	2000	1,035
2	Effects of nitrogen sources on cell growth and lipid accumulation of green alga *Neochloris oleoabundans*	Applied Microbiology and Biotechnology (Germany)	2008	821
3	High-quality biodiesel production from a microalga *Chlorella protothecoides* by heterotrophic growth in fermenters	Journal of Biotechnology (Netherlands)	2006	775
4	High-value products from microalgae—their development and commercialization	Journal of Applied Phycology (Netherlands)	2013	645
5	Lipid accumulation and CO_2_ utilization of *Nannochloropsis oculata* in response to CO_2_ aeration	Bioresource Technology (United Kingdom)	2009	577
6	Characterization of a microalga *Chlorella* sp. well-adapted to highly concentrated municipal wastewater for nutrient removal and biodiesel production	Bioresource Technology (United Kingdom)	2011	482
7	Omega-3/6 fatty acids: Alternative sources of production	Process Biochemistry (United Kingdom)	2005	442
8	Amino Acid Absorption by Arctic Plants: Implications for Plant Nutrition and Nitrogen Cycling	Ecology (United States)	1994	438
9	Progress in the biological and chemical treatment technologies for emerging contaminant removal from wastewater: A critical review	Journal of Hazardous Materials (Netherlands)	2017	413
10	Mapping of picoeucaryotes in marine ecosystems with quantitative PCR of the 18S rRNA gene	FEMS Microbiology Ecology (United Kingdom)	2005	393

### Country and Collaboration

**Figure 2:**
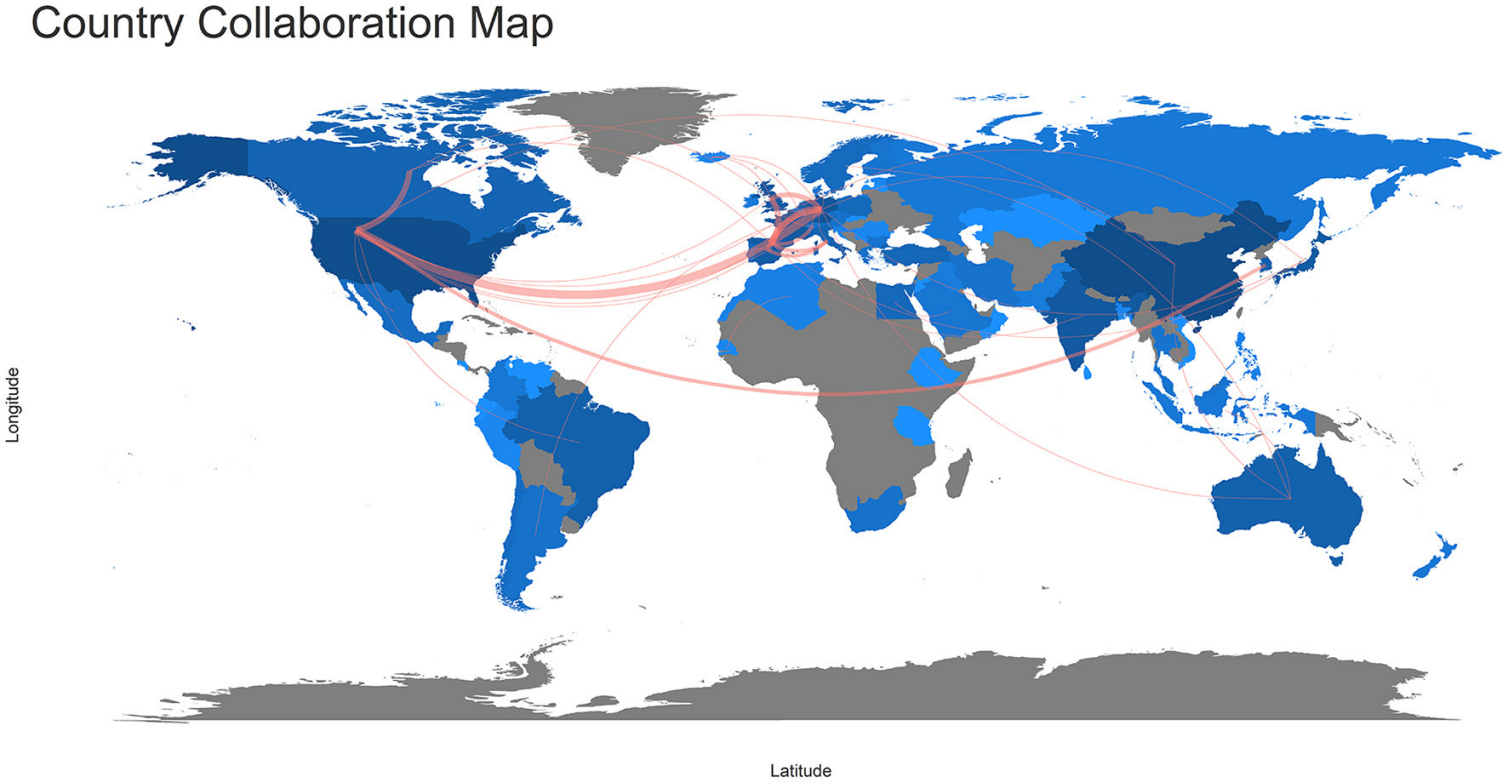
Country collaboration map of microalgae-fungi consortia publications.

China, the United States (USA), and Germany were the most prolific countries publishing about microalgae-fungi consortia, counting 360, 344, and 155 publications each. They were followed by France (148), India (113), Japan (103), Spain (101), the United Kingdom (UK) (94), and Brazil (70). Figure 2 presents the country collaboration map. The thicker the red line between the countries, the stronger teamwork between them. European countries showed intense collaboration among them and with the USA. Accordingly, the USA (9,803 citations), China (5,580), and France (3,215) were the countries that accumulated more article citations without taking into account the publication language.

**Figure 3:**
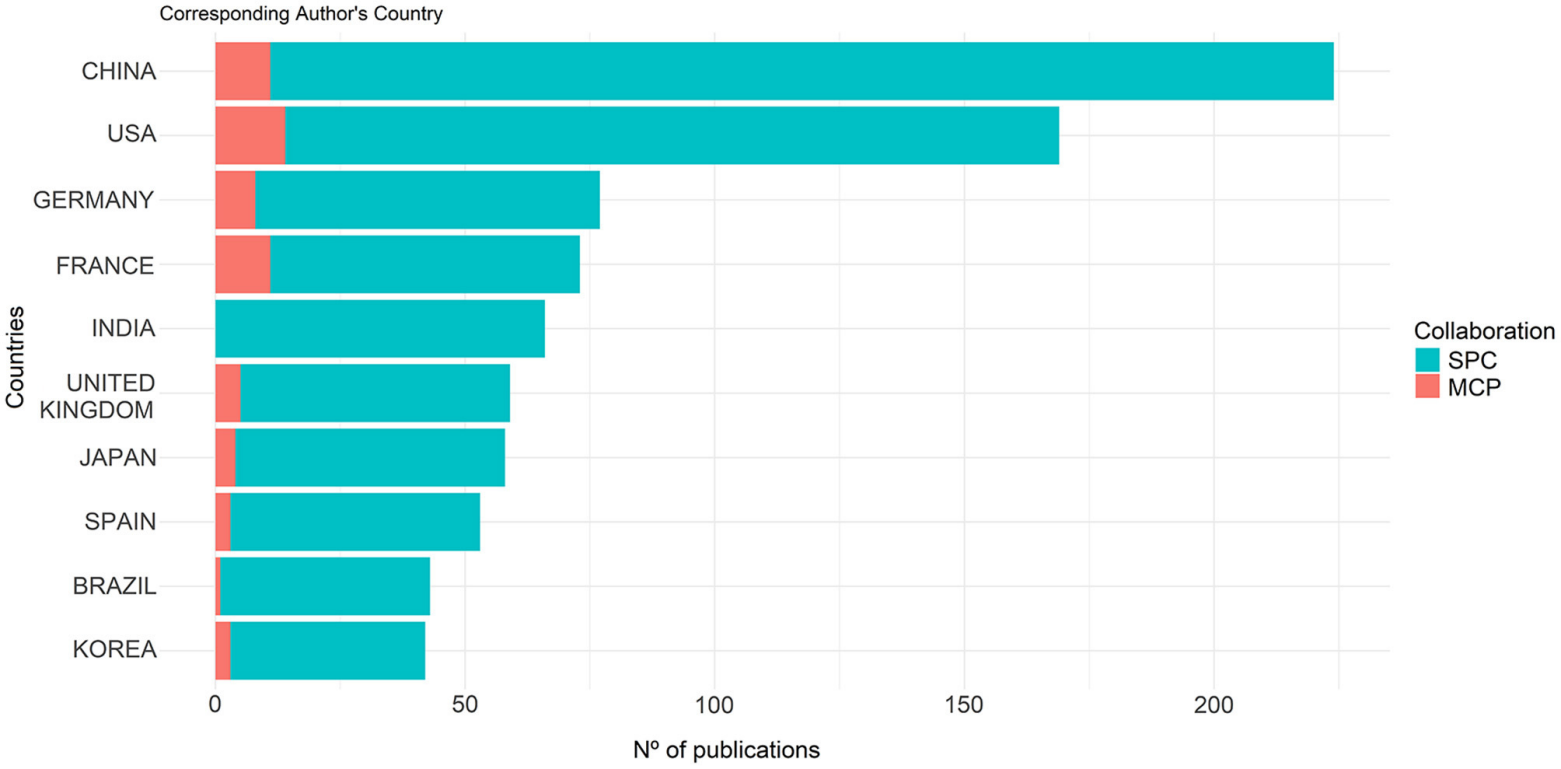
Single and multiple countries' publications on microalgae-fungi consortia.

Despite the collaboration between countries, it was evidenced that amongst the 10 most prolific countries, the single country publications (SCP) still count a higher number of articles than the ones on which multiple countries (MCP) have collaborated (Figure 3). In this regard, the country that most collaborated internationally was the USA, even though it was the 2nd in the number of total publications. India was the only country that did not present any MCP.

### Authors and Institutions

**Figure 4:**
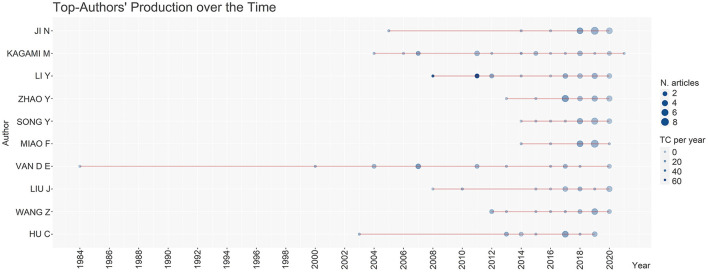
Publication timeline of the 10 most-prolific authors on microalgae-fungi consortia.

The articles were signed by 4,974 authors, an average of 3.3 authors per article. Single authored articles summed 5.9%, and 71 distinct authors individually signed these. Of the 10 most-prolific authors, 8 were from China, and 2 were from the Netherlands. A timeline of the 10 most-prolific authors is displayed in Figure 4. Most of the authors started to publish on the subject after 2000.

Chinese institutions also figured between the top 10 ranks of the most-productive institutions. The National Cheng Kung University was the 1st in several publications (25 articles), followed by the University of Chinese Academy of Science (21). The USA appeared twice on the rank occupying the 5th (University of Minnesota−17) and the 9th (University of California−15) positions. France represented Europe in the 3rd position (Centre National de la Recherche Scientifique – 19) and Italy in the 10th position (University of Naples Federico II−15). No countries from Central and South America appeared on the rank, like Asia and Oceania.

### Sources

**Figure 5:**
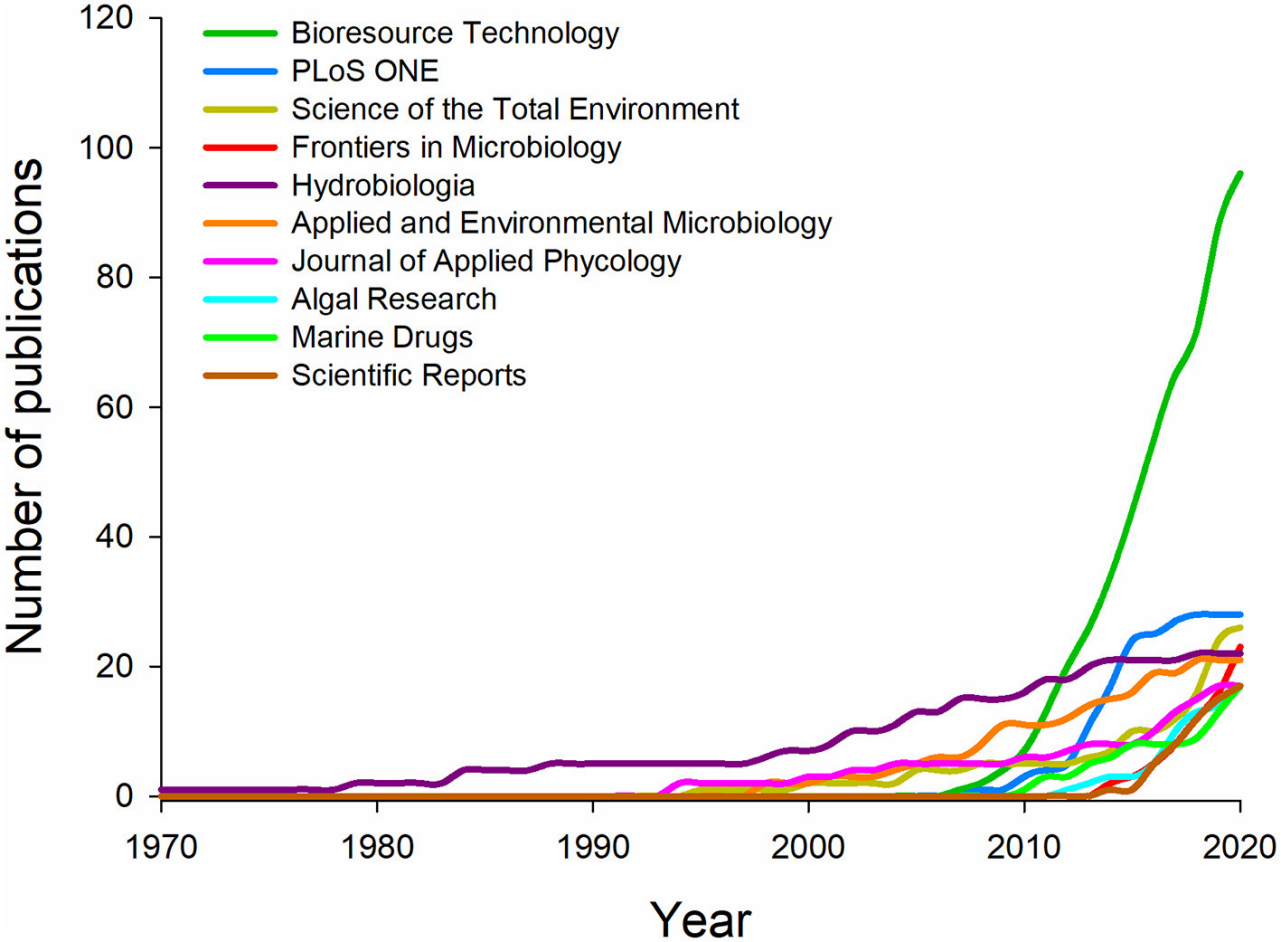
Microalgae-fungi consortia publication increased in the 10 most-productive journals in the last 50 years.

Articles were cataloged in 575 journals, but 62.7% of these only published 1 article each. Bioresource Technology was, by far, the most-productive journal with 96 articles published, followed by PLoS ONE (28) and Science of the Total Environment (26) (Table 2). The top 10 journals published 19.6% of the articles. The increase in publications by the 10 most-productive sources over the last 50 years is shown in Figure 5. All journals increased their publication rates in the last decade, detaching Bioresource Technology, PLoS ONE, and Frontiers in Microbiology, which exhibited leverage on the number of publications after 2010.

**Table 2:** The 10 most-productive journals in microalgae-fungi consortia.

	**Source**	**Articles**	**Country**
1	Bioresource technology	96	United Kingdom
2	PLoS ONE	28	United States
3	Science of the total environment	26	Netherlands
4	Frontiers in microbiology	23	Switzerland
5	Hydrobiologia	22	Netherlands
6	Applied and environmental microbiology	21	United States
7	Journal of applied phycology	18	Netherlands
8	Algal research	17	Netherlands
9	Marine drugs	17	Switzerland
10	Scientific reports	17	United Kingdom
	Other (475)	1,167	

### Keywords

**Figure 6:**
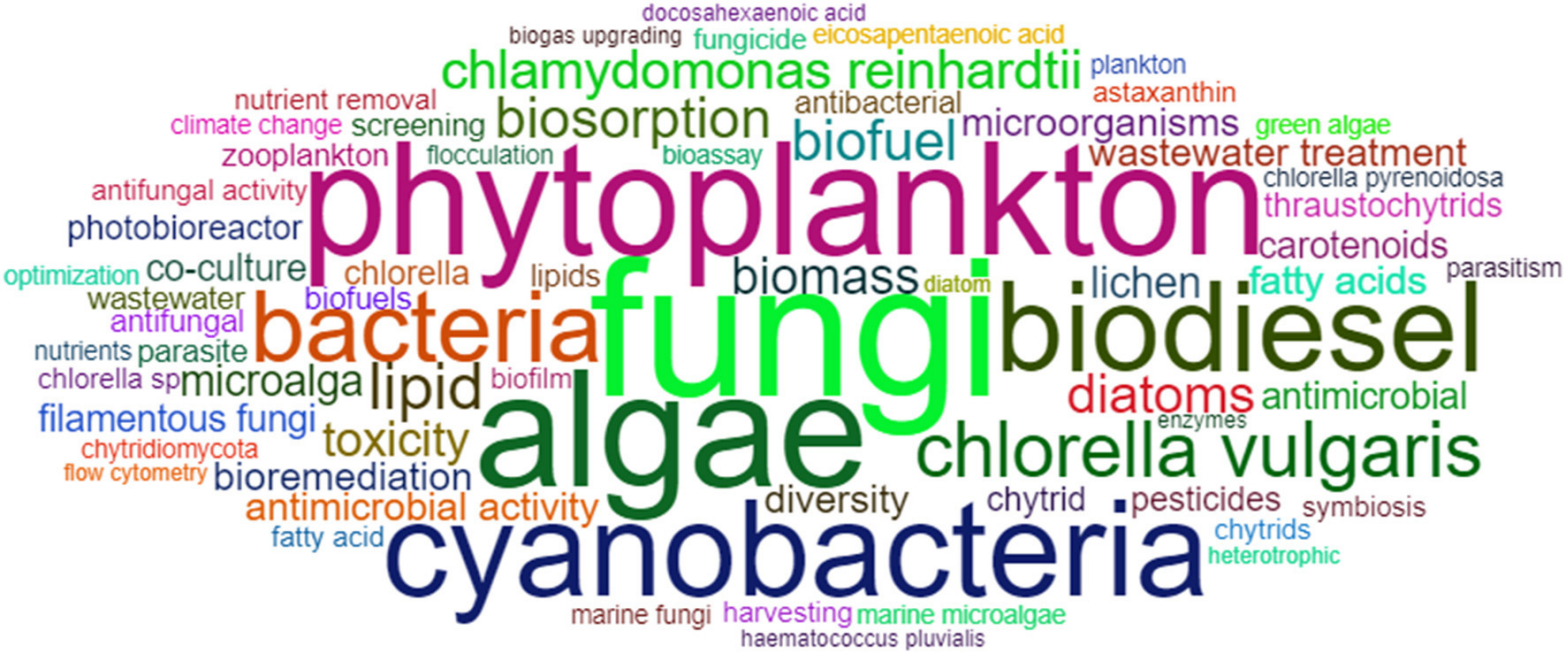
Most-cited keywords of microalgae-fungi consortia publications.

To reveal the main publication trends and subjects, we analyzed 3,794 keywords. A word cloud showing the most-cited keywords is presented in Figure 6. The “x” following the numbers after the keywords mentioned means the times they were cited. The word Microalgae was cited 204 times, followed by Fungi (67 ×), Algae (57 ×), Phytoplankton (55 ×), and Cyanobacteria (47 ×). To complete the top 10 ranks of the most-cited keywords, Biodiesel (44 ×), Bacteria (35 ×), Lipid (34 ×), Biofuel (31 ×), and *Chlorella vulgaris* (27 ×) emerged from the 1,452 articles. After biodiesel, biogas-related words (e.g., biogas upgrading and anaerobic digestion) were mentioned 26 times and ranked in the 64th position of the most-cited.

Summing the 2nd most-cited keyword (Fungi) and Filamentous fungi (27th position), this group of organisms counted 80 mentions in the articles. In Biology, the association between algae and fungi can be called Lichen (15 ×) (Srinuanpan et al., 2018), and their co-culture (21 ×) is a promising topic in Biotechnology. Filamentous fungi (the most cited genus in this investigation were *Trichoderma* sp. and *Aspergillus* sp.) have been used to assist microalgae biomass harvesting (27 ×) through bioflocculation (25 ×) (Zhou et al., 2013), seen as a low-cost technique (Jiang et al., 2021) for bioproducts and biofuel production (Zhou et al., 2012).

Another important step for the utilization of microalgae biomass is the pre-treatment (11 ×) performed by fungal enzymes (11 ×) that break the microalgae hard cell wall (Mahdy et al., 2016; Rai et al., 2021). The two most-cited microalgae species in the keyword analysis were also the most well-studied (Garrido-Cardenas et al., 2018). The *C. Vulgaris* and *Chlamydomonas reindhardtii* (19 ×) are characterized by different aspects. At the same time, the first is the most-studied species and presents a hard cell wall composed of cellulose, hemicellulose, and glycoproteins (Passos et al., 2016), while the cell wall is unprovided on the latter (Mahdy et al., 2016).

**Figure 7:**
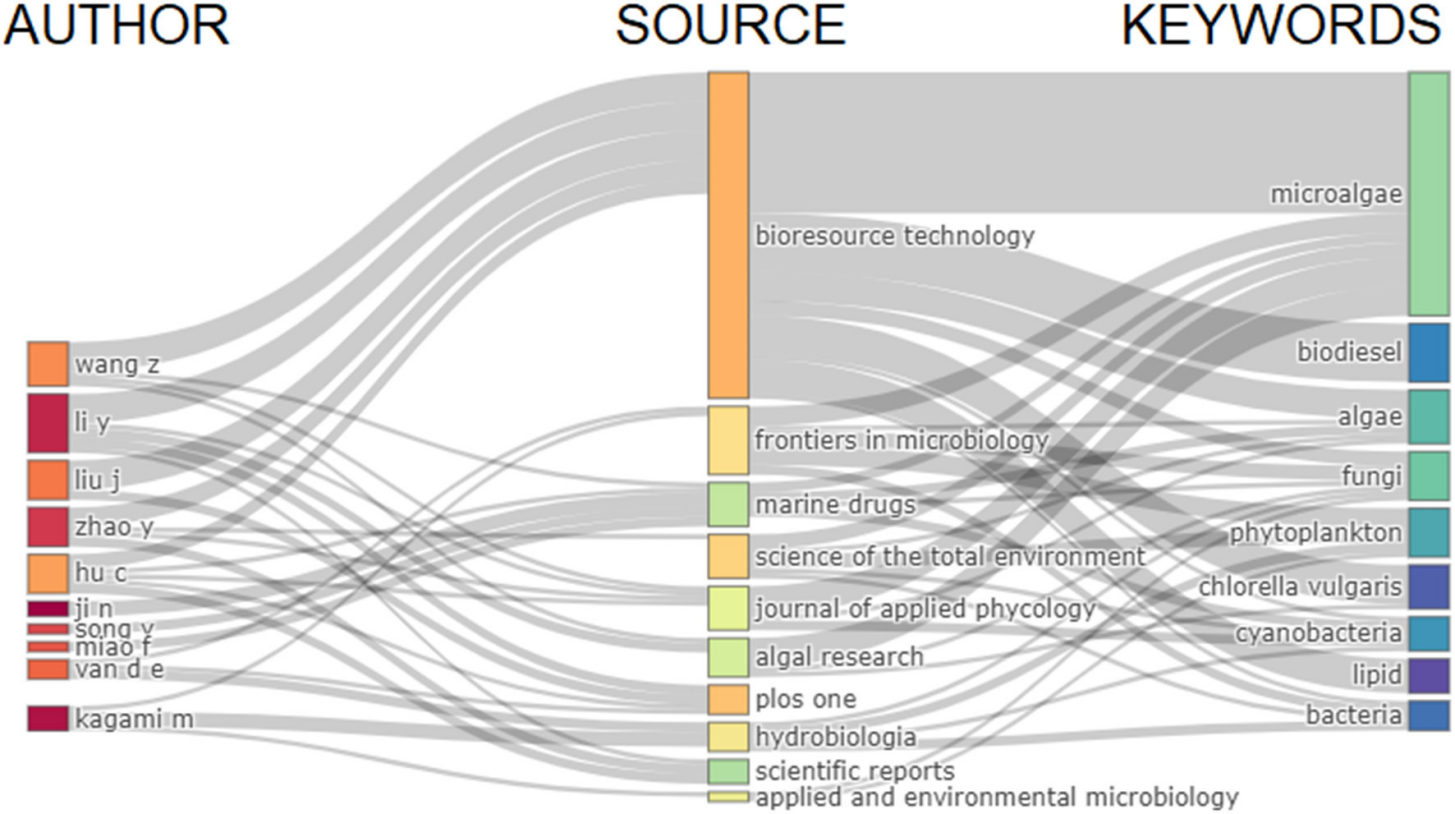
The connection between authors, journals, and keywords.

As evidenced by the keyword analysis, we found a bias for biofuel research in the articles, especially biodiesel and biogas. The connection between the most prolific authors, journals, and most-cited keywords is displayed in Figure 7. For example, confirming Nabout et al. (2015) and Rumin et al. (2020), microalgae, biodiesel, lipid, and *C. Vulgaris* were keywords strongly connected to the journal Bioresource Technology, which focused on biotechnology and bioenergy [also discussed in Zhang et al. (2018)]. On the contrary, phytoplankton was linked to Hydrobiologia, a journal focused on ecological studies.

## Discussion

Economic interests, biorefinery, and the research around microalgae-fungi consortia have raised since 1950 (Cheng et al., 2020), and during the oil crisis in the 1970 decade microalgae was considered a potential source for energy production (Baicha et al., 2016). Notwithstanding, the number of publications found in this investigation was smaller than in other research focused only on microalgae, which counted from 20,000 and 70,000 publications (Garrido-Cardenas et al., 2018; Rumin et al., 2020). Different from studying each group of organisms individually, we expected a lower number of publications while investigating this microbial consortium. Comparing, a review focused on microalgae fungal-assisted bioflocculation included only 18 articles (Nazari et al., 2020).

Since the technology outbreak, globalization, and software development in Computer Science, the English language has become part of political and economic confluence. In the twentieth century, high investments in education were made by developed nations, and the hegemony of the English language was certified as the Science language (Hamel, 2007). Not surprisingly, English dominated the publications about microalgae-fungi consortia in our results (96.3%). It confirms that even for researchers, whose mother tongue is not English, publishing in English is an important factor in ensuring their results' wide distribution and comprehension.

Publishing the results is the main goal for researchers that worked hard to confirm or refute their hypothesis. The 1,452 articles found in our results counted almost 50 thousand citations until 2020. We observed that the publication numbers have grown over the years and also the article citations (see Figure 1). It can be considered evidence of the subject's relevance among the scientific community and the advances in biotechnological development of microalgae-fungi co-culture. Furthermore, these citations represent a good means to spread the new findings published, enriching the discussion of the upcoming publications worldwide (Larsen and von Ins, 2010).

The collaboration between countries reinforced the significance that microalgae-fungi consortia are gaining. Scientists are summing efforts to develop new knowledge in the field and exchange their findings, technologies, and research results with other researchers and society. Even though the most published country was China, the USA showed more collaboration with different countries on their publications. In general science, the USA has been the leading country in several publications since the 1990 decade (King, 2004), and here we also stated its collaboration strength. Garrido-Cardenas et al. (2018) found a similar pattern of collaboration between the USA and European countries while mapping the microalgae research worldwide.

Despite that, China was the country with the most prolific authors. The country has implemented high investments in education and funding; many Chinese researchers left the country to study abroad and then came back, helping to sustain the country's rapid economic growth (King, 2004). Also, the increase in investments in Research & Development (R&D) in China has contributed to the rising of Science (Gonzalez-Brambila et al., 2016). In 2017, the Chinese government invested 2.15% of the Gross Domestic Product (GDP) in R&D. This amount is only lower than that found in South Korea, Japan, Germany, the USA, and France's R&D investments (Chiarini et al., 2020).

Azadi et al. (2017) reported that the number of scientists and engineers in China has tripled in 20 years (between 1990-and 2010). Similar to this study, Cheng et al. (2020) evidenced that half of the most-fruitful institutions were also from China. Finally, Garrido-Cardenas et al. (2018) found that 4 of the most-productive institutions were Chinese, including the Chinese Academy of Sciences and the National Cheng Kung University, which also ranked in our results. It is notable the progress and the innovation capacity of Chinese universities and authors over the last years (Gonzalez-Brambila et al., 2016).

Different from the biotechnological profile of Chinese authors, 2 of the most prolific authors were from the Netherlands and linked to the Netherlands Institute of Ecology. Nabout et al. (2015) stated that the term phytoplankton is broadly studied in Ecology, and the Netherlands figures as the 7th most-contributive country in algae ecology research (Konur, 2020c) and also on bioenergy/biofuels publications (Konur, 2020a). European countries have strong participation in research involving microalgae and phytoplankton, registering 33% of the world's publications (Rumin et al., 2020). Likewise, the USA and China published around 3,000 articles each (Garrido-Cardenas et al., 2018).

To reinforce the tendency of using the keyword “microalgae” in biotechnology publications, Bioresource Technology was the journal that published the most about microalgae-fungi consortia. In the same way, Garrido-Cardenas et al. (2018) searched for “microalga^*^” and the same source was also ranked as the most prolific. Similar to the observed in this study, Rumin et al. (2020) ranked 4 of the most-productive journals focused on Ecology, while Bioresource Technology occupied only the 5th position when searched for “microalgae” and “phytoplankton” keywords.

When the keyword “phytoplankton” was not included in the search, Konur (2020a) ranked only 2 of the 10 most-productive journals ranked in this study (Bioresource Technology and Journal of Applied Phycology). It confirmed that the keyword “phytoplankton” used in our search has influenced the publication outputs presenting results related to Ecology besides Biotechnology. Otherwise, when the search was done only for the genus *Chlorella* sp., Cheng et al. (2020) ranked 7 of the 10 most-productive journals in this research, evidencing the importance of this microalgae genus to current investigations about algae in Biotechnology.

Algae biofuels have turned into one of the most rising topics in the renewable energy scenario. Nowadays, China is the country that most contributed to the crescent propagation of this thematic (Azadi et al., 2017), differently from what happened in the past 10 years, when the USA and European countries were responsible for 70% of the total publication in this research field (Adenle et al., 2013). In our search, the word “biofuel^*^” appeared as a trend among microalgae-fungi consortia research, especially focused on “Biodiesel” and “Biogas.” The energy production of microalgae-based biofuels can be 100 times higher than most crops used in the first and second generations of biofuels (Kirrolia et al., 2013).

It is known that more than a few types of biofuels can be produced from microalgae, e.g., lipids can be converted to biodiesel through transesterification; carbohydrates can be used for bioethanol production through fermentation, biogas is produced *via* anaerobic digestion, and finally, biohydrogen through biophotolysis (Bahadar and Bilal-Khan, 2013; Kiran et al., 2014; Zhu et al., 2017; Sankaran et al., 2018). In all these processes, fungi can cooperate in harvesting microalgal biomass through bioflocculation. However, this is one of the most expensive steps, costing up to 20–30% of biofuel production costs (Molina-Grima et al., 2003), and is still a bottleneck on microalgae-based biofuel biorefinery (Gerde et al., 2014).

Biodiesel was first reported on databases in 1991, while the microalgae-based biodiesel concept appeared in 1993 (Ma et al., 2018). The high lipid content of microalgae is one of the most attractive features of its use in biofuel biorefinery (Kiran et al., 2014; Hwang et al., 2016). Considering the required cultivation area, some microalgae species can produce 300 times more oil than some edible crops from the first generation of biofuels (Alam et al., 2015). Even though liquid biofuels have been more explored than gaseous (Yaoyang and Boeing, 2013). Anaerobic digestion of algae for biogas production, in its turn has been an established technology being studied and utilized for more than 60 years from the 1950 decade until nowadays (Golueke et al., 1957; Zabed et al., 2020). Biogas is a biofuel commonly derived from the second generation of biomass for biofuels (Azadi et al., 2017), but it has considerably emerged as a hot topic in the microalgae-based biofuel scenario (Córdova et al., 2018).

Figuring between the most-cited species in this investigation, *C. vulgaris* is one of the most studied and cultivated microalgae species worldwide for biofuel production (Garrido-Cardenas et al., 2018). This genus was widely studied during the 2nd World War as a nutritional supplement. Then, its ability to remove recalcitrant compounds through bioremediation started to be investigated, and the high lipid content is an extremely valuable compound to the biofuel industry (Cheng et al., 2020). Filamentous fungi also appeared on the top-cited keywords, and the most species applied to the bioflocculation of microalgae are *Aspergillus* sp., *Trichoderma* sp., *Penicillium* sp., *Rhizopus* sp., and *Mucor* sp (see Chu et al., 2021).

Rajendran and Hu (2016) defined this synthetic association called “Mycoalgae”; the co-cultivation of microalgae-fungi is advantageous in many biorefinery processes, reducing the energetic and financial costs of the harvesting process. Also, this topic has gained emphasis in the scientific community, reflected by the increase in the number of publications, likewise on the citation parameters, and the range broad of countries, institutions, and authors investigating this consortium. Finally, the analysis of the keywords revealed the main trends related to the topic, highlighting the terms associated with biofuel production, especially biodiesel and biogas.

## Conclusion

Through the analysis of 1,452 articles, an increase in the number of publications is observed between 1950 and 2020, especially highlighted from the year 2006 onward, the same period where an increase for microalgae-derived products took place. In addition to the publication trends, the languages of the publications were also evaluated, and 96.3% of the articles are in English. All the articles summed 49,052 citations; the 10 most-cited are published in English, while 2 are are related to biodiesel manufacture. That confirms the trend for biofuel research sought in this investigation. The country publishing the most is China (360), followed by the USA (344), and far behind in the 3rd position is Germany (155).

On the other hand, the USA has the highest number of multiple countries' collaboration. Unlike those observed in other microalgae research, the most participative institutions are Chinese (1st and 2nd positions), while the others are from the USA and Europe. The most-prolific authors are Chinese, except for 2 authors from the Netherlands. The results evidence that China occupies a very prominent spot in microalgae-fungi research. The sources publishing the most about this consortium are distributed between Biotechnology (as Bioresource Technology) and Ecology (e.g., Hydrobiologia) fields, confirming the bias of using the terms microalgae and phytoplankton in the search. The analysis of the keywords also indicates the trends for biofuel production expected in this investigation. Biodiesel is the 6th most cited keyword, confirming that biodiesel is the most studied biofuel type, like lipid, the main microalgae byproduct used for biodiesel production. Also, but in a distant position, Biogas (64th) is another type of biofuel that emerged from the keyword analysis. Finally, coupled with a range of filamentous fungi species, *Chlorella* sp., ranks as the most-studied microalgae genus in the keywords.

These findings can collaborate on understanding how microalgae-fungi co-cultivation is being investigated and guide future research on exploring the potential of this consortium for biotechnology, bioproducts, and biofuels. Through this effort, we conclude that there is a gap in exploring new microbial-consortia actors, including fungi and microalgae, that are not yet bioprospecting. Moreover, applications for carbon-mitigation emissions using microalgae-fungi consortia are welcome.

## Data Availability Statement

The original contributions presented in the study are included in the article/supplementary material, further inquiries can be directed to the corresponding authors.

## Author Contributions

AL-M: conceptualization (lead), writing—original draft (lead), formal analysis (lead), investigation (lead), and writing—review and editing (equal). SX-S: conceptualization (supporting), writing—review and editing (equal), investigation (supporting), and financial resource (equal). LD-S: review and editing (equal) and financial resource (equal). SC: conceptualization (supporting), investigation (supporting), writing—review, editing (equal), and financial resource (equal).

## Funding

AL-M scholarship was provided by the Fundação de Amparo à Pesquisa do Estado de Goiás, process n° 202110267000890. This study was financed in part by the Coordenação de Aperfeiçoamento de Pessoal de Nível Superior - Brasil (CAPES) - Finance Code 001 (Convênio n° 817164/2015 CAPES/PROAP).

## Conflict of Interest

The authors declare that the research was conducted in the absence of any commercial or financial relationships that could be construed as a potential conflict of interest.

## Publisher's Note

All claims expressed in this article are solely those of the authors and do not necessarily represent those of their affiliated organizations, or those of the publisher, the editors and the reviewers. Any product that may be evaluated in this article, or claim that may be made by its manufacturer, is not guaranteed or endorsed by the publisher.
